# Metagenomic Approaches to Assess Bacteriophages in Various Environmental Niches

**DOI:** 10.3390/v9060127

**Published:** 2017-05-24

**Authors:** Stephen Hayes, Jennifer Mahony, Arjen Nauta, Douwe van Sinderen

**Affiliations:** 1 School of Microbiology, University College Cork, Cork T12 YT20, Ireland; stephen.hayes@umail.ucc.ie (S.H.); j.mahony@ucc.ie (J.M.); 2 APC Microbiome Institute, University College Cork, Cork T12 YT20, Ireland; 3 Friesland Campina, Amersfoort 3800 BN, The Netherlands; Arjen.Nauta@frieslandcampina.com

**Keywords:** virome, phage, marine, microbiota

## Abstract

Bacteriophages are ubiquitous and numerous parasites of bacteria and play a critical evolutionary role in virtually every ecosystem, yet our understanding of the extent of the diversity and role of phages remains inadequate for many ecological niches, particularly in cases in which the host is unculturable. During the past 15 years, the emergence of the field of viral metagenomics has drastically enhanced our ability to analyse the so-called viral ‘dark matter’ of the biosphere. Here, we review the evolution of viral metagenomic methodologies, as well as providing an overview of some of the most significant applications and findings in this field of research.

## 1. Introduction

Viruses infecting bacteria, or bacterio(phages), are presumed to represent the most abundant biological entities on earth [1]. They exist wherever bacterial life is found [2], with millions of phages found in every drop of seawater [3], and the human gut is estimated to contain between 10^8^ and 10^9^ virus-like particles (VLPs) per gram of faeces [4,5], of which many are undoubtedly phages. Since their initial discovery in 1915 by Frederick Twort, followed by the realisation in 1917 by Felix d’Herelle that phages had the potential to kill bacteria [6], phages have been at the cutting edge of molecular biology research, both as model systems and as potential biological tools for the manipulation of bacterial genomes [7]. Numerous studies have explored the role of phages in various ecosystems, and it has thus become apparent that phages exert their influence across every aspect of life. For example, cholera toxin, the causative agent of many symptoms of cholera, is encoded by a temperate bacteriophage or prophage [8], as are the virulence factors in various pathogens causing bacterially-derived food poisoning and diphtheria [9,10].

Given their ubiquity in the environment, it is perhaps unsurprising that bacterial evolution is to a large degree driven by phages [11,12], facilitated by recombination with or the integration of prophages [13] or by evolutionary responses to evade lytic phage infection [14]. Thus, bacteriophages are a core part of the ecosystem, modulating microbial nutrient cycles, community structure, and long-term evolution [15,16,17]. Despite this, phages are often marginalised, if not completely ignored, in many studies, and this omission may thus result in conclusions that ignore a crucial part of the picture [18]. Therefore, even though phage genomes are orders of magnitude smaller than bacteria and thus easy to sequence, less than 2700 double-stranded DNA (dsDNA) virus and retrovirus genomes are deposited in NCBI (National Center for Biotechnology Information) databases, compared to almost 90,000 prokaryotic genomes (as of February 2017). Consequently, a full understanding of the scope of virus-host interactions is lacking [19], and current estimates state that the field of virology has possibly explored less than 1% of the extant viral diversity [20]. However, with in silico, culture-independent techniques becoming available in recent decades, this is beginning to change. In this review, we assess the range of methods employed in viral metagenome analyses, while we also outline the limitations and advances in the field. Furthermore, we will discuss the range of environmental niches where such approaches have been successfully implemented to improve our understanding of viral communities and their perceived impact on microbial landscapes.

## 2. Culture-Dependent vs. Culture-Independent Methods of Bacteriophage Study

For over a century, culture-based methods have been the primary approach to detect and isolate phages [20]. This involves cultivating hosts and isolating individual phages from plaques using methods such as double agar assays. However, such approaches carry significant limitations due to the recalcitrance of the majority of microbes to cultivation under laboratory conditions [21]. It is currently predicted that up to 99% of microorganisms are unculturable [22,23]. Moreover, even in the presence of a culturable host, phage identification and isolation may remain difficult [24], as not all phages are capable of forming plaques and there is evidence that many successfully infect in the absence of discernible plaque formation [25,26]. Further factors such as pseudolysogeny (the stalled development of a bacteriophage in a host cell) [27] and differences between laboratory-based assays and phage–host interactions in nature [28], with many phages for example requiring their host to be in a specific growth phase to successfully infect [29], only serve to increase the difficulty of studying phages. Thus, while a wealth of information has been gained from these methods, it is clear that culture-independent methods are vital to further our understanding of the diversity of phages and their role and impact in various environmental niches.

### Culture-Independent Methods of Bacteriophage Study

The first culture-independent methods arose in the 1980s as an approach to characterise microbial diversity via DNA sequencing [30,31,32]. These early techniques utilized 16S ribosomal DNA (rDNA) as a common marker gene since it is universally present in bacteria and archaea [33,34,35]. However, the limitations of such approaches, combined with the lack of universally conserved markers in phages [36], has led to the development of alternative technologies for the culture-independent study of bacteriophages, including randomly amplified polymorphic DNA (RAPD) PCR [37], flow cytometry [38], electron microscopy [3,39,40], single virus genomics [41], and viral tagging [42] (Table 1). Studies employing electron microscopy showed that viruses are far more abundant in the oceans than previously predicted and by inference in several other ecological niches. These techniques testify to the rapidly increasing and evolving range of approaches to the culture-independent study of previously inaccessible viral communities. Furthermore, it highlights that their use either individually or as part of a complementary approach can provide insights into the population composition and genetic diversity of environmental viral samples. However, by far the most successful method that has arisen for the culture-independent study of viral communities is metagenomic analysis.

## 3. Metagenomics

The term metagenomics was first coined in 1998 [58] and is defined as the direct sequencing and analysis of all genetic material recovered from an environmental sample [59]. There are two primary approaches used for the metagenomic study of uncultured microbial populations; shotgun metagenomics, which involves the sequencing of the entire nucleic acid compliment of a sample [60], and marker gene amplification metagenomics, typically using the 16S ribosomal RNA gene [61]. The optimal method to be used varies according to the goals of a study and the resources available. Full shotgun metagenomics is far more costly and time-consuming but, due to the unrestricted sequencing of all genomes in the sample, will provide far more information, while 16S metagenomics is largely restricted to taxonomic composition of the bacterial/archaeal population of the sample but is much more rapid and less costly [62]. Thus, using these techniques, both qualitative and quantitative analysis of uncultured microbial communities has now become possible [63]. This versatility, coupled with the emergence in the new millennium of widely available high-throughput sequencing (HTS), has resulted in metagenomics becoming the most effective and comprehensive approach for the genomic analysis of uncultured microbial populations [24]. As a result, the number of published metagenomic studies has risen explosively from 11 in 2002 to over 10,000 in 2017 (using metagenomics as a search term in the PubMed search engine), while there are now more than 8000 metagenomic datasets (of which over 4600 are publicly available) deposited in the Integrated Microbial Genomes and Microbiomes (IMG/M) system, a public comparative resource for both sequenced genomes and metagenomic datasets [64]. Far from simply producing massive volumes of sequencing data, these metagenomic studies are continually yielding novel information such as genomic linkage between function and phylogeny and evolutionary profiles of community function and structure [59], as well as facilitating the discovery of novel genes and enzymes [65,66,67,68].

## 4. Viral Metagenomic Sample to Sequence Pipeline

**Figure 1:**
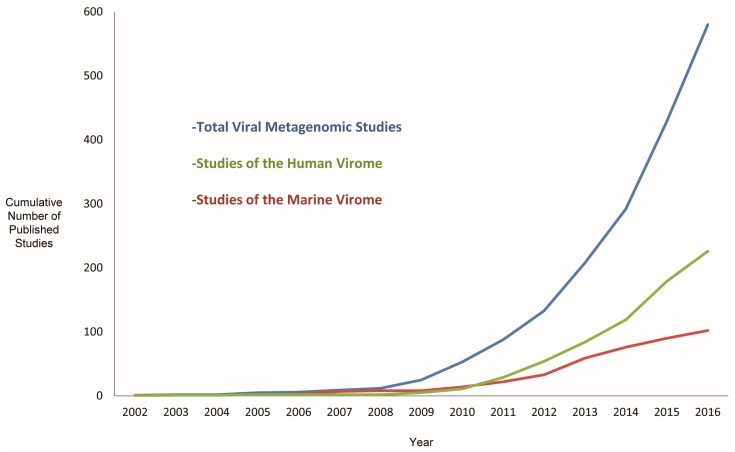
Graph illustrating the large increase in the publication of viral metagenomic studies from the initial study of Breitbart et al. [73] in 2002 to the end of 2016. The total cumulative number of studies is represented in blue. The number of metagenomic studies of the human virome is represented in green, and studies of the marine virome in red. The number of studies in each case was determined via Pubmed search.

The growing success of microbial metagenomics, coupled with the increasing awareness of the vital role of viruses in nature, has resulted in attention quickly turning towards the application of metagenomics to the field of virology. The challenges of applying metagenomics to viral samples are many: the lack of universal marker genes, meaning full shotgun metagenomics is required; contamination with bacterial DNA, which is far more abundant than viral, and the consequential difficulties in separating these during sequence analysis [69]; the vast diversity of virus types in nature, making the isolation, sequencing, and assembly of an unbiased viral metagenome extremely difficult [70]; and a lack of viral sequence in databases, making comparative genomics of limited value, to name but a few. However, viruses also possess a number of characteristics favourable to metagenomic analysis. For example, their small size permits the effective removal of cellular debris by centrifugation and/or filtration, allowing rapid purification of phages in a diverse array of samples. The characteristic buoyant densities of viruses facilitate their selective purification via cesium chloride gradient [71] (although it must always be remembered that this will select against VLPs outside the densities examined, biasing the resulting population [72]). Additionally, the comparatively small genome size of viruses is well suited to sequencing techniques, provided nucleic acids of sufficient quality and quantity can be isolated [18]. Thus, as metagenomic techniques have advanced, the field of viral metagenomics has expanded significantly (Figure 1). Since the first application of viral metagenomics to uncultured marine samples in 2002 [73], virome (the nucleic acid complement of all viruses in a population) studies have been applied to a wide range of environments and locations including freshwater, seawater, soil, industrial fermentations, and the guts of humans and many other organisms. Due to the diversity of environments, there is no single, one-fits-all method that can be employed, with protocols requiring sample/source-specific adaptations. However, studies generally involve a number of key steps: (i) viral particle purification; (ii) nucleic acid extraction, (iii) high-throughput sequencing of purified viral nucleic acids, and (iv) bioinformatic analysis and interpretation of sequence data. While the application of metagenomics to the study of phages is the primary focus of this review, phages are generally isolated from a sample in congruence with eukaryotic viruses, except if the desired phages could be separated from eukaryotic viruses in the early stages of a metagenome isolation using methods such as density gradients. Indeed, most virome studies aim to characterise all viruses, only distinguishing phages during the final analysis of sequence data. Thus, the following viral metagenomic pipeline provides a broad overview of techniques for the generation of viral metagenomes, which is equally applicable to both phages and eukaryotic viruses.

### 4.1. Viral Particle Extraction

**Figure 2:**
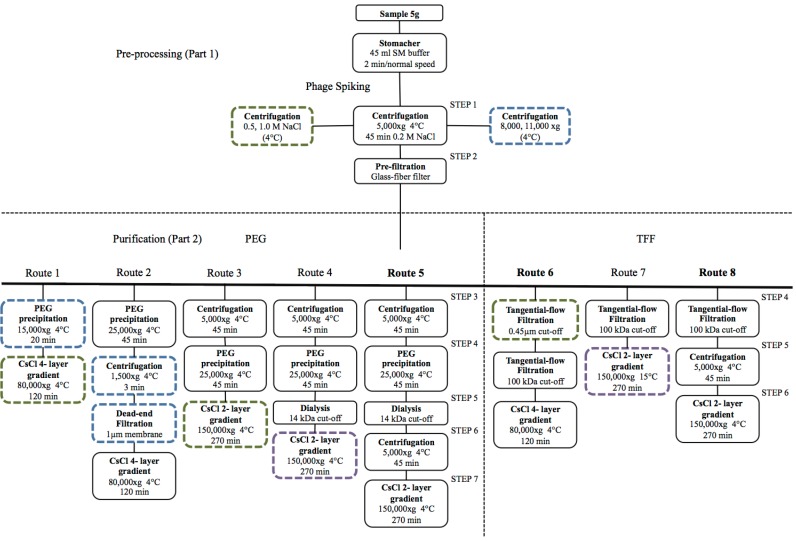
Optimization of the extraction of phages from the human gut for viral metagenomic analysis. In this study, samples were ‘spiked’ with a set titre of known phages, and the efficiency of recovery of these phages was monitored throughout a number of extraction protocols. Part 1 (pre-processing) involves the suspension/dissolution of phages from the samples and the removal of large particles. Part 2 (phage purification) then removed lower molecular weight impurities and microbial cells. Boxes with non-continuous borders represent steps that were deemed unsuitable for phage extraction, either due to large losses of spiked phages or impure samples. Green bordered boxes represent steps that resulted in impure samples, as assessed by visual inspection and transmission electron microscopy (TEM). Blue bordered boxes indicate steps at which >50% of spiked phages were lost. Purple bordered boxes signify steps that failed to remove microbial contamination. Two main purification routes were optimized polyethylene glycol (PEG) precipitation and tangential flow filtration (TFF), and routes were diverted into new extraction routes (i.e., from route 1 to route 2, etc.) until the highest recovery of spiked phages was reached. This optimisation resulted in the purification of greatly increased numbers of phage particles and much higher quantities of DNA in comparison to previous protocols [74]. Reprinted with permission from Castro-Mejía et al. [74].

The purification of VLPs from an environmental sample is the initial step of any virome project and arguably the most critical. Techniques must strive to produce VLP samples that both qualitatively and quantitatively represent the diversity of the population, thus allowing the linkage of metagenomic observations to the original population [24]. The basic steps indicated below can be adapted to suit the requirements of the sample of interest: (i) recovery of VLPs within the sample, (ii) VLP purification and concentration, and (iii) optional final purification via cesium chloride gradient [24,74,75,76,77,78]. The extraction and purification of bacteriophages from the human gut for viral metagenomic analysis has recently been optimised [74], and the extraction protocols used in this study are outlined in Figure 2. By optimising the basic extraction steps previously mentioned, this study succeeded in greatly increasing the number of phage particles and the quantity of viral DNA obtained and produced two optimised extraction protocols, which are easily modifiable and thus in principle applicable to almost any environmental sample [74].

Beyond the general approaches, specialised methods catering to particular environments have been developed such as the flocculation, filtration, and resuspension (FFR) method, which uses an iron-based flocculation and subsequent resuspension of virus-containing precipitates and which boasts a 90% recovery rate of marine viral particles [79,80].

### 4.2. Nucleic Acid Extraction

The next step in the preparation of a sample is the extraction and purification of its viral nucleic acid content. This step must yield nucleic acids of sufficient purity and concentration for downstream library construction and sequencing [59]. Before this step, a DNase treatment is generally performed to eliminate contaminating cellular DNA, the presence of which can drown out the sequences recovered from viral genomes, while also creating difficulties in downstream analysis [69]. However, due to the diverse range of virus types, the nature of this treatment will be highly dependent on the aims of the researcher and the focus of the study. Nucleic acid extraction is then generally performed using one of a range of commercially available kits [76], although it is important to note that studies in bacteria suggest that the choice of kit can have an influence on the community structure produced by HTS [81], which could in turn result in inaccurate or biased conclusions being drawn [82]. After viral DNA extraction, PCR analysis employing 16S/18S rRNA gene-based primers may be useful to assess the presence of bacterial host DNA, offering a semi-quantitative measure of microbial contamination [83], and quantitative PCR (qPCR) can be used to provide a more accurate estimate of host contaminants.

### 4.3. Library Preparation and Sequencing

The advent of next generation sequencing (NGS) has resulted in the emergence of rapid, affordable, and high throughput methods such as Illumina MiSeq and Ion Torrent sequencing [84], making metagenomic sequencing far more accessible. In preparation for sequencing, libraries are prepared from the isolated viral DNA, following fragmentation to suitable lengths for the specific sequencing platform(s) to be used whilst minimizing sample loss and preventing the introduction of bias [84]. Varying fragmentation techniques are used depending on the sequencing platform, with an in-depth summary of suitable methods and their advantages and disadvantages discussed in [85]. Fragmentation frequently results in single-stranded DNA (ssDNA) ends, which require repair in preparation for the next step in sequencing, dsDNA adaptor ligation. Once the adaptors are ligated, they serve as primer sites during the sequencing reaction and may also contain a barcode to allow the sequencing of pooled libraries in a single run.

Amplification is commonly one of the final steps in library preparation before sequencing, creating ca. 1000 copies of the DNA to be read by the sequencer [85]. This step is especially crucial to virome studies as, though viruses are much more abundant than their hosts, their genomes are orders of magnitude smaller than those of microbes [24]. The earliest metagenomic amplification technique was linker-amplified shotgun libraries (LASLs) [25]. LASLs consist of randomly sheared cDNA fragments ligated to known adapter sequences for PCR amplification, which are then cloned into plasmid vectors and Sanger sequenced [73]. Whole genome amplification using methods such as multiple displacement amplification (MDA), which utilises the polymerase of Φ29, were developed to improve throughput using NGS platforms [25]. However, these systems have been shown to not only introduce a systematic bias related to the preferential amplification of single-stranded and circular DNA templates, which is particularly relevant in the case of viral metagenomics [86], but also non-predictable and random biases [87]. These combined biases skew the taxonomic representation of a community, resulting in non-quantitative metagenomes [88,89], thereby preventing comparative analyses.

Improved library preparation methods have been developed (Table 2), including linear amplification for deep sequencing (LADS) [90] and the Nextera fragmentation and adapter ligation methods [91]. Nextera is an extremely rapid two-step method in which the simultaneous fragmentation and tagging of genomic DNA using modified transposition in a process designated as ‘tagmentation’ [91] is followed by a reduced cycle PCR to add adaptors. However, Nextera has been demonstrated to introduce amplification bias, preferentially amplifying genomic regions of low GC content [91]. Additionally, Nextera requires a minimum of 50 ng of starting material, which can be difficult for certain virome samples [24], although the newer iteration, Nextera XT, has reduced this requirement to 1 ng, with one study of a microbial metagenome even lowering this to 50 pg of input DNA [92]. The LADS protocol, developed for Illumina Sequencing, has emerged as an attempt to circumvent GC bias by replacing the PCR step with a transcription step [90]. However, LADS requires significant expertise and is particularly labour-intensive [25]. Despite recent advances, an optimization of the original linker amplification (LA) method [93] by Duhaime et al. in 2012 [94], may provide the most quantitative, next-generation-sequence-ready DNA and can be adapted for use on the Illumina, 454 or Ion Torrent sequencing platforms [24].

Improved library preparation techniques are still emerging, including the previously mentioned Nextera XT, multiple annealing and looping-based amplification cycles (MALBAC), and NuGEN’s Mondrian microfluidic workstation used in conjunction with the NuGEN Ovation library prep kit. MALBAC reduces amplification bias and increases coverage by utilising a semi-linear amplification method [96], while the Mondrian approach automates much of the library preparation protocol. The impact of these three methods, along with template quantity, on the metagenomic output obtained from a mock microbial community from as little as 1 pg of DNA has recently been assessed [97]. It was found that template quantity in all three methods had a significant impact on the revealed community composition, highlighting that unbiased amplification techniques are beyond current capabilities and represent an area that will need further improvement [97]. However, the ability to perform metagenomic sequencing with reduced DNA quantities (as compared to previous protocols) represents an especially relevant advance in the field of viral metagenomics.

Once a DNA library has been prepared, sequencing is performed, primarily utilising one of the three main NGS platforms; Illumina (currently the most popular [62]), Roche 454 (discontinued in 2016), and Ion Torrent PGM (for a review on sequencing technologies, see [98]). Following sequencing, quality control steps are performed as recently reviewed [84] to ensure that the sequence data is ready for analysis. These quality controls include ensuring the adequate sequencing coverage of each sample and the removal of rare reads, which may represent sequencing errors or contamination and thus cause overestimation of the protein richness of a virome [80].

### 4.4. Analysis

While continuing advances in sequencing technology have opened up a multitude of opportunities in the field of viral metagenomics, the enormous amounts of data produced by NGS has also resulted in a major challenge; quality analysis and the processing of sequence data. The primary constraining factor regarding the effective bioinformatic analysis of viromes is the previously discussed absence of universal gene markers in viral genomes, meaning that the detection of viral reads is limited to their alignment against reference viral sequence databases. As of February 2017, the NCBI virus genome database contained just over 2000 phage genomes, with nearly half of those derived from just four genera of bacteria, which have been studied in detail due to their clinical relevancy (*Mycobacterium*, *Enterobacteria*, *Pseudomonas*, and *Staphylococcus* [84]). It is widely accepted that virtually all bacteria suffer from phage predation; however, just eight of the 29 known bacterial phyla with cultured isolates have sequenced phage representatives [99]. Therefore, it is clear that vast data exists that is inaccessible by current comparative analysis. The lack of sequence identity typically results in viral metagenomes containing between 60% and 99% of sequences, which possess no significant homology to sequences in current databases [89]. However, this high proportion of ‘unknown’ sequences also presents the greatest opportunity, with a treasure trove of uncharacterised sequences.

The taxonomic composition of a sequenced viral metagenome is often analysed by the alignment of the virome against reference databases using BLAST (Basic Local Alignment Search Tool), or BLAST-based programs such as MG-RAST (MetaGenomic-Rapid Annotation using Subsystem Technology) [100], MetaPhyler [101], or CARMA [102], which is a time-consuming process. Alternatively, rapid fast *k*-mer algorithms may be applied to reduce the time associated with such analyses [84,89]. These algorithms are more than 50× faster than alignment-based approaches [103] and are incorporated in programs including CLARK (CLAssifier based on Reduced *K*-mers) [104] and USEARCH [105]. However, these more rapid methods typically require heavy computing power (>128 Gb of Random Access Memmory (RAM)) [104] and are thus restricted to comparisons against reference databases such as Refseq, which, given the modest proportion of viral representation in these databases, limits the level of annotation possible. For example, Hurwitz et al. (2013) compared the annotation of the Pacific Ocean Virome (POV) dataset, originally obtained employing BLASTx alignments against all known proteins at the genus and family level [106] against taxonomic data obtained via a re-annotation using CLARK [84]. The authors found that 1.12% and 0.96% of reads matched regions in bacterial and viral genomes, respectively, using CLARK, in contrast to 4.01% and 6.87% matches against bacterial and viral proteins using BLASTx. This example serves to highlight that there is currently a persistent trade-off in the field of viral metagenomics between speed and accuracy.

### 4.5. Bioinformatic Tools for Viral Metagenome Analysis

In order to circumvent the large demands for computer processing power of these methods, a number of online resources and tools have become available, making metagenomic analysis more accessible to the uninitiated. These computational pipelines have been designed for the purpose of analysing the composition of metagenomic datasets; in the case of viromes, this means that the abundance and types of viruses present in a sample can be defined. These include virome-specific programs such as VIROME (Viral Informatics Resource for Metagenome Exploration) [107], Metavir [108], and VMGAP (Viral MetaGenome Annotation Pipeline) [109] and also more ‘generalist’ pipelines including those previously mentioned programs incorporating BLAST based analysis. These pipelines are generally utilizing ORF (Open reading frame)-finding algorithms, which predict coding sequences followed by subsequent comparison with protein databases. A recent study by Tangherlini et al. [110] involved an in-depth comparison of these tools for the analysis of the taxonomic composition of both simulated and actual benthic deep-sea viral metagenomes. This study confirmed translated BLAST (tBLASTx) as the most reliable tool for the accurate analysis of viral diversity, followed by the Metavir tool. Furthermore, the authors highlight that, as with all steps in the viral metagenome process, the choice of bioinformatic tool can significantly influence the obtained findings and derived conclusions [110].

In addition to these tools, all based upon sequence comparisons to reference databases, numerous similarity-independent methods have arisen in order to circumvent the lack of sequence similarity in current databases [111]. The primary tool designed for this purpose remains PHACCS (Phage Communities from Contig Spectrum), which provides estimates of the richness, evenness, and abundance of the most abundant viruses in a viral metagenome [112], based on the principle that the most abundant virotypes (taxonomic classification based on a percentage identity threshold rather than phylogenetic markers) will more likely be assembled into large contigs [111]. Other reference-independent tools include MaxiPhi [113], which analyses inter-sample diversity between two samples, and crAss [114], which facilitates the simultaneous cross-assembly of all samples in a data set.

These tools offer just a sample of those available, and the range of bioinformatic tools ready for use in the analysis of viral metagenomes has recently been reviewed [115,116]. Moreover, new tools are continually emerging, such as VirSorter [117] and MetaPhinder [118], both designed for the detection of viral sequences in metagenomic data; VirusSeeker, released in early 2017 (mainly focused on eukaryotic viruses, though it does incorporate bacteriophage analysis in the pipeline [119]); and the iVirus community resource, which provides access to a range of viral metagenomic tools and datasets [120]. Thus, as methods improve, the discrepancies and biases introduced by these programs will hopefully be overcome.

## 5. Current and Potential Areas of Interest for Viral Metagenomics

By applying the workflow outlined in Section 4 to the sample of interest, it is theoretically possible to perform viral metagenome analysis on virtually any sample. Indeed, a plethora of studies have already been performed on an array of environments, and some of the dominant niche areas are discussed below.

### 5.1. Marine Viral Metagenomics

Since the pioneering study of Breitbart et al. in 2002 [73], marine phage genomics has been at the forefront in the field of viral metagenomics. Oceans cover over 70% of the Earth’s surface, produce over half of the oxygen in the atmosphere, and absorb the most carbon dioxide from it. Driving these energy cycles are the marine microbes, which constitute more than 90% of the living biomass in the sea. Considering that viruses kill roughly 20% of this biomass each day [16], driving the so-called ‘viral shunt’ (the role of viruses in the transformation of living biomass into dissolved organic matter) [57], it is clear that marine bacteriophages play a critical role in the biosphere. Consequently, a decade after the above-mentioned first marine virome study, a large-scale, consortia-driven study [106] of the marine virome culminated in the development of the previously mentioned POV.

**Figure 3:**
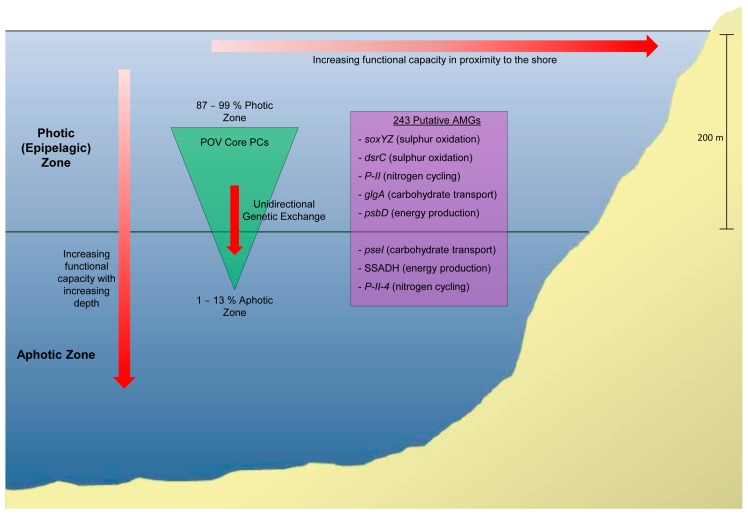
Functional richness of the ocean virome as determined by the Pacific Ocean Virome (POV) [106] and the Global Ocean Virome (GOV) [125] datasets. Analysis of functional diversity revealed that the functional richness of viral communities decreased in surface communities as distance from the coast increased and increased in the ocean as depth increased. It was also found that core PCs (protein clusters) of the POV (i.e., those present in all samples) are enriched in the photic zone relative to the aphotic zone, indicating unidirectional genetic exchange from surface waters to the deep ocean. Additionally, 243 putative auxiliary metabolic genes (AMGs) were identified, examples of which can be seen for each zone.

The POV dataset consists of 32 near-quantitative dsDNA viromes collected from samples at various locations, depths, and seasons and has led to many novel insights [57]. To organise the ‘unknown’ sequences, which comprised the vast majority of viral sequences obtained, protein clusters (PCs) were formed using an approach originally applied to microbial metagenomics [121]. Protein clusters are groups of ORFs grouped together by sequence similarity [122], and this resulted in the creation of over 450,000 PCs to aid in the mapping of future viromes [106]. In addition, by performing rarefaction analysis of the PCs obtained from different locations/depths/seasons, the community diversity of the viral populations was compared. From this, it was demonstrated that the functional capacity of viral communities varied according to depth, season, and distance from the shore (Figure 3), with the greatest richness (not to be confused with abundance, richness simply measures the number of species in a community without considering the population size of each) observed in areas near to the shore and in the aphotic zone of the ocean during winter [106]. Furthermore, it was found that core PCs (those present in every sample) were enriched in the photic zone relative to the aphotic zone, possibly indicating unidirectional genetic exchange from the surface to deep oceans, most probably facilitated by viral particles descending rapidly in aggregated biomass via vertical flux [123]. Finally, the rarefaction curves of POV PCs lowered the predicted number of marine PCs to 0.5–1.3 million and the global virome to 3.9 million PCs [124], far lower than the two billion distinct viral proteins previously predicted [1].

The POV also provided the first in-depth, large-scale survey of the presence of Auxiliary Metabolic Genes (AMGs) in marine viruses. AMGs are host-derived genes present in phage genomes, and their encoded proteins are suggested to augment the metabolism of infected hosts at key metabolic bottlenecks, facilitating and enhancing the production of new viral particles [126]. Sequence data from the POV greatly advanced the repertoire of known AMGs and demonstrated that AMGs are far more diverse than previously thought [127]. Virtually all genes encoding functions that are required for carbon metabolism were found in the virus population, as well as genes involved in amino acid production and energy production and genes involved in other cellular functions such as motility and transport [123]. Thus, the POV has provided a wealth of information and continues to be consolidated and expanded by emerging studies.

A number of other large-scale investigations have been performed [57], and in 2016 the Global Ocean Virome (GOV) was released [125]. The GOV is comprised of 104 virome datasets (both surface- and deep-ocean) sampled during the Tara Oceans and Malaspina research expeditions [128,129], providing a global map of abundant, dsDNA marine viruses. In this study, viral contigs were clustered based on co-occurrence and nucleotide signature to form ‘viral populations’, roughly equivalent to viral species. Using this method, 15,280 viral populations were identified, and rarefaction analyses indicated that, as a result of the GOV, sampling of dsDNA viral communities in the epipelagic zone, or surface layer, of the ocean is now nearing completion [125]. Virus populations, along with the publicly available phages and archaeal viruses, were then categorised into Viral Clusters (VCs), roughly corresponding to viral genera [130]. This produced 1259 VCs, of which 658 were exclusive to the GOV, and 209 others were contained GOV sequences, indicating a doubling of known phage and archaeal virus genera. Subsequently, the global abundance of each VC was analysed, and it was observed that only 38 of 867 VCs were abundant in more than one station. Interestingly, of the 38 ‘abundant’ VCs, only 20 contained previously known (either from viral isolates or environmental sequencing) viral sequences, while 18 were completely unreported. Thus it is clear that, even in the case of some of the most globally abundant viruses, much is still unknown about them.

The GOV consortia then went further, and sought to link this viral data to microbial hosts. The linking of viral sequence data to host strains has long been a major challenge in the field of viral metagenomics [57], although a number of new methods have emerged. Such methods include physical methods such as ‘viral tagging’ [42] and sequence-based methods utilising similarity approaches. Similarity based approaches include similarity searches between viral and microbial genomes [131,132]; linking viral genomes and their host via clustered regularly interspaced short palindromic repeats (CRISPR) spacers [133]; and comparing viral and host genome nucleotide signatures [130]. These methods were applied to the GOV, which led to host-range predictions for 392 VCs [125].

Finally, the prevalence of AMGs in the sequence data was investigated, and 243 putative AMGs were identified, of which only 95 were previously known [123]. These AMGs included genes with reputed roles in sulphur and nitrogen cycling, with analysis revealing many genes vital to these pathways in epipelagic viruses [125].

The POV and GOV datasets are not definitive representatives of the marine virome due to the limitations and inherent biases of current viral metagenomic techniques and equipment. The above-mentioned studies were dominated by the examination of dsDNA viruses, thus leaving ssDNA viruses underrepresented, an issue which is currently one of the most pressing areas of concern in the field of viral metagenomics [70,134]. However, the POV, GOV, and many other studies of the marine virome have unquestionably demonstrated the potential of viral metagenome analysis of environmental samples, and exponentially expanded our knowledge of viral communities in the oceans while providing critical reference frameworks for future studies.

### 5.2. Human Viral Metagenomics

Human metagenome studies have assessed the complex microbial communities associated with the mouth, gastrointestinal tract, lungs, and skin, among other areas. Additionally, human studies have attempted to define correlations between microbial composition and the subject’s age, health status/disease states and lifestyle. Among these, the human gut is undoubtedly the most extensively characterised and was thoroughly reviewed recently [135]. While such studies provide an array of data pertaining to the bacterial landscapes in these niches, it is imperative to simultaneously assess the role of phages in shaping these landscapes. In view of this, a plethora of recent virome studies of a variety of human surfaces/organs and the impact of different demographic and environmental conditions have been undertaken and will be discussed herein.

#### 5.2.1. Virome of the Skin and Oral Cavity

The epidermidis of human skin serves as the primary protective barrier to the external environment and the surface and natural crevices of the skin are host to a variety of microbes. A recent study highlighted that, consistent with other studies, the total metagenome of skin in healthy individuals exhibits temporal stability, while the virome is subject to much greater variability [136]. Bacterial species including *Propionibacterium*, *Staphylococcus*, and *Corynebacterium* were identified in the subjects’ skin in this study, while the viruses recovered through virome analysis of isolated VLPs were dominantly either unclassified or identified as *Staphylococcus* phages. The presence of high levels of seemingly novel phage sequences in this niche exemplifies the high degree of viral dark matter that is present and highlights that current knowledge on the skin virome represents merely the ‘tip of the iceberg’ of the genetic diversity of phages in the biosphere. The majority of phages recovered from this environment are dsDNA viruses, and this apparent dominance is likely owing to the temperate nature of the majority of these isolates. Most metagenome studies employ Illumina-based sequencing technologies as they produce high sequence coverage, and they are relatively inexpensive. However, the application of PacBio SMRT (single molecule real time) sequencing was recently applied to produce ‘finished quality’ genomes of skin metagenome samples and resulted in the identification of a previously uncharacterised *Corynebacterium simulans* phage–host genome combination [137]. To assess the potential diversity of the strains of this species in the sample, HiSeq datasets were simultaneously generated and revealed a dominant strain within the population. Therefore, while high throughput sequencing technologies are the method of choice, long-read technologies/hybrid approaches may be useful in the evaluation and reconstruction of complex microbial and viral communities.

It is reported that the human oral cavity is host to six billion bacteria and up to 35 times as many phages [138]. In congruence with the findings in the skin environment, the viromes of the oral cavity are suggested to primarily contain temperate phages, with 10% of viral reads possessing integrase homologues in a study of the saliva of five healthy subjects [139]. Viral reads with homology to viruses of *Veillonella*, *Streptococcus*, and *Megasphaera* were identified in this study, and, in addition to the identification of lysogeny-related functions, virulence factors were also abundant, which are asserted to present a reservoir of pathogenic potential/conversion in the human oral environment. It is predicted that the viral communities in the oral cavity are persistent, which is likely a reflection of the stability of host bacterial communities in this environment [138]. Furthermore, phages that exist in such complex communities encounter significant competition, which places them under a high level of evolutionary pressure to adapt to their host if the latter is changing and to ensure the success of the host so as to secure their own persistence.

#### 5.2.2. Virome of the Lungs

The human lung microbiome has been the focus of several recent studies with implications for understanding and advancing treatment of a variety of pulmonary (and other) diseases. The relationship between the microbiome of the upper and lower respiratory airways is unclear and represents an area of growing interest in the research community. Furthermore, the impact of diseases such as cystic fibrosis (CF) and human immunodeficiency virus (HIV) infection on the lung microbiome has recently received justified research scrutiny [140,141,142]. In contrast to the array of studies on the lung microbiome, relatively few studies have looked at the lung virome, although the value of such studies has recently been highlighted [143]. Therefore, it is likely that an increasing number of lung virome studies will be undertaken in the near future. Viral metagenomic analysis of samples from the respiratory tract of lung transplant patients identified an increase in the relative abundance and complex populations of human-associated anelloviruses, which are small circular, non-enveloped, ssDNA viruses [144]. In a recent study of the metagenome and virome of the respiratory tracts of CF patients, viral reads displaying sequence relatedness to phages infecting typical CF pathogens including *Streptococcus*, *Burkholderia*, *Mycobacterium*, *Enterobacteria*, and *Pseudomonas* were identified [145]. Surprisingly, the phage profiles between distinct patients were quite similar, while, in contrast, greater diversity was observed among the microbial communities. This suggests that the recovered phage sequences had multiplied on persistent host bacteria and were thus stable, while, in contrast, transient microbial populations were less conserved and their infecting phages (if present) would presumably be less abundant than the persistent colonisers in CF patients. This finding is remarkable among virome analysis studies and highlights the condition/environment-specific nature of viromes, particularly when considering disease states.

#### 5.2.3. Virome of the Human Gut

The microbiota of the human intestinal tract is undeniably one of the most intensely studied microbiomes and is estimated to be constituted by up to 10^14^ bacterial cells [146]. Studies assessing the effect of age [147,148,149], geographical location [150], diet [151,152], and health status [153,154,155], among other factors, on the human gut microbiota have been undertaken. Similar to other fields of metagenomics, there is an increasing awareness that it is essential to evaluate the presence and potential impact of (pro)phages on the microbiome; therefore, there is a growing number of studies in this area. Recently, the prophage-related sequences identified on the genomes of 38 bifidobacterial strains were extracted and used to identify the presence of related sequences in the metagenomic data of infants [155]. *Bifidobacterium* sequences were identified in the data relating to 20 of the 173 infants, and (pro)phage sequences largely with *Siphoviridae* genomic organisation were additionally identified with relatedness to phages of *Actinobacteria*, *Firmicutes*, and Gram-negative bacteria. The virome of the human gut is influenced by the health status of the individual, and patients with Crohn’s disease and ulcerative colitis were shown to exhibit ‘abnormal’ viromes, with a much greater level of phages belonging to the *Caudovirales* family [156]. The viromes were also condition-specific, highlighting the need for data pertaining to individual disease states. Furthermore, while the gut virome of individuals over time may not change dramatically in most cases, it is highly individual-specific [157,158]. The individuality and condition-specific nature of the gut virome and its sensitivity to environmental factors consolidate the requirement for extensive and representative sampling to provide robust data.

One of the most intriguing findings from the many human gut virome studies was the identification of the so-called ‘crAssphage’ [159]. This phage and its related sequences are highly abundant among the viromes of all human faecal metagenome datasets [160]. This phage, which was until very recently unknown to exist, represents just one example of the necessity for the examination of what currently constitutes viral dark matter and highlights the possibility of defining and understanding the vast array of phage–host interactions that may exist and influence our gut microbial landscapes.

### 5.3. Potential Applications

Microbial metagenomics has long been recognised as possessing the capability to significantly influence industrial biotechnology [161] from functional screening of metagenomic sequences for novel enzymes [162] to increasing our understanding of the dynamics of various biological processes such as food fermentations, thus facilitating improved efficiency, product quality, and profitability [63]. The field of viral metagenomics possesses similar potential, although its applications to this end remain understudied and very much in their infancy.

Novel enzyme discovery is chief among these applications. Bacteriophages have long been a rich source of enzymes [7], but the majority of the most commonly used enzymes in laboratories are still derived from a handful of cultivable phages such as T4, T7, λ, and Φ29 [163]. These phages have yielded crucial enzymes such as T4 DNA ligase, utilized in virtually all laboratory ligations during cloning, and numerous DNA polymerases [164]. However, considering the abundance of phage sequences that continue to be isolated either via culture-dependent or metagenomic methods, as well as the genetic diversity and enzyme-richness of these sequences, it is clear that the functional potential of bacteriophages remains greatly under-exploited. Functional viral metagenomics has the potential to rectify this, facilitating the discovery of technologically useful enzymes. For example, a single 2 Mb bacterial genome possesses a single DNA polymerase 1 gene, while up to 20–40 *pol1* genes can be found in 2 Mb of viral metagenomic sequence [163]. Aside from the identification of suitable hosts for the expression of potential enzymes, however, the primary challenge preventing the realisation of this potential is the difficulty in annotating viral metagenomes [164]. Nonetheless, methods are continually improving, and recent studies have identified numerous thermostable DNA polymerases from viral communities in hot springs in Yellowstone National Park (YNP), California, and Nevada [164,165].

The discovery of novel enzymes is not limited to replication-related genes. Bacteriophage lysins are highly evolved enzymes produced by phages which cleave the bacterial cell wall during the final stages of the lytic cycle to facilitate the release of phage particles. Due to their function, attention has turned to these enzymes and their potential use as novel antimicrobials [166], including their application as food preservatives and as therapeutic agents against human pathogens [167], where the host-specific activity of phage lysins prevents non-target negative effects in addition to circumventing antibiotic resistance. As a result, attention has turned towards the application of functional viral metagenomics to the discovery of novel phage lysins [168].

These examples offer a snapshot of the potential of functional viral metagenomics to serve as a platform to unlock the wealth of useful enzymes that is undoubtedly present in the vast viral sequence space. Indeed, as the annotation of viral sequences continues to improve, the discovery of novel enzymes will increase considerably.

## 6. Conclusions and Future Perspectives

The continual improvement of technology and techniques to minimise the introduction of biases and the skewing of produced population structures is the primary challenge facing the field of viral metagenomics. The challenges remain many and varied, but as the methods approach a level of quantitative rigour capable of producing faithful representations of environmental viral communities, viral metagenomics can transition from a tool of observation and description to a means of prediction and application. These advances will also increase confidence in the validity of viral genomes identified purely through metagenomic sequencing, leading to the acceptance of these sequences as bona fide viruses and their inclusion in formal ICTV (International Committee on Viral Taxonomy) viral taxonomy, a process about which discussion has already begun [169]. The identification of the widespread existence and abundance of crAssphage in the human gut indicates the existence of previously unknown and uncharacterised viral entities and highlights the wealth of undiscovered data that may exist. Viral metagenomics is poised to vastly increase our knowledge of viral dark matter and to further elucidate the fundamental role viruses play in every aspect of the biosphere.

## Figures and Tables

**Table 1:** Selection of culture-independent methods for the study of bacteriophages.

Method	Description	Limitations
Gene marker-based studies [43,44,45,46,47]	Utilise marker genes, ranging from major capsid proteins to photosynthesis related genes, to study the diversity of viruses in a sample.	Lack of universal viral gene limits the focus of studies to particular phage genera [48]; cannot provide quantitative analysis [24].
Randomly Amplified Polymorphic DNA (RAPD) PCR [37]	Uses short, random primers to amplify fragments of environmental DNA of assorted sizes. Provides a rapid, rudimentary comparison of viral diversity.	Limited inferences possible; difficult to reproduce results due to high sensitivity of the technique to reaction conditions [49].
Electron microscopy [3,39,40]	Allows enumeration of uncultured viruses, particularly in marine samples. Accuracy and speed improved by epifluorescent microscopy [50,51,52,53].	Limited to observation of morphologies and rough estimates of quantity of viral particles; no sequence data generated.
Flow Cytometry [38,54,55]	Rapid enumeration of viral particles in a sample via their staining with highly fluorescent nucleic acid dyes followed by counting via flow cytometry.	Limited to estimations of quantity; no sequence data generated or morphology information.
Single virus genomics [41]	Enables isolation and complete genome sequencing of single viral particles. Involves sorting of single viruses by flow cytometry, followed by genome amplification via multiple displacement amplification (MDA) and whole genome sequencing.	Does not provide community-wide view of viral population.
Viral Tagging [42,56]	Allows study of phage–host interactions by fluorescently labelling phages and using them to ‘tag’ their host. Phages inject labelled genomes into their host, rendering the bacteria fluorescent. Potential hosts are then sorted via fluorescence-activated cell sorting (FACS).	Requires a culturable host, extensive optimisation required for each new host [57].

**Table 2:** Required nucleic acid quantities, advantages, and drawbacks of being commonly employed for virome library preparation.

Method	Nucleic Acid Quantity	Advantages	Drawbacks
Multiple displacement amplification (MDA) [95]	1–100 ng	Rapid and high-throughput	Introduces both predictable and stochastic biases
Linear amplification for deep sequencing (LADS) [90]	3–40 ng	Low levels of bias introduced, resulting in near-quantitative metagenomes	Low throughput, requires significant expertise
Linker amplified library construction [94]	>10 pg	Remains the most quantitatively accurate method, requires minimal nucleic acid input	Low throughput, requires significant expertise
Nextera XT (Illumina)	50 pg	Rapid, combines fragmentation and tagging of DNA into single 5 min ‘tagmentation’ step	Slight sequence-dependent biases at low nucleic acid input levels [92]
